# Design, Synthesis, and Renal Targeting of Methylprednisolone-Lysozyme

**DOI:** 10.3390/ijms21061922

**Published:** 2020-03-11

**Authors:** Xingquan Pan, Fei Xie, Dian Xiao, Xinbo Zhou, Junhai Xiao

**Affiliations:** National Engineering Research Center for the Emergency Drug, Beijing Institute of Pharmacology and Toxicology, Beijing 100850, China; panxingquan1991@163.com (X.P.); xiefei0058@163.com (F.X.); be_xiaodian@163.com (D.X.)

**Keywords:** methylprednisolone, lysozyme, renal targeting, proximal tubular epithelial cells

## Abstract

Methylprednisolone (MP) is often used in the treatment of various kidney diseases, but overcoming the systemic side effects caused by its nonspecific distribution in the body is a challenge. This article reports the design, synthesis, and renal targeting of methylprednisolone–lysozyme (MPS–LZM). This conjugate was obtained by covalently linking MP with the renal targeting carrier LZM through a linker containing an ester bond, which could utilize the renal targeting of LZM to deliver MP to renal proximal tubular epithelial cells and effectively release MP. The reaction conditions for the preparation of the conjugate were mild, and the quality was controllable. The number of drug payloads per LZM was 1.1. Cell-level studies have demonstrated the safety and endocytosis of the conjugate. Further pharmacokinetic experiments confirmed that, compared with that of free MP, the conjugate increased the renal exposure (AUC_0–t_) of active MP from 17.59 to 242.18 h*ng/mL, and the targeting efficiency improved by approximately 14 times. Tissue and organ imaging further revealed that the conjugate could reach the kidneys quickly, and fluorescence could be detected in the kidneys for up to 12 h. This study preliminarily validates the feasibility of a renal targeting design strategy for MPS–LZM, which is expected to provide a new option for improving kidney-specific distribution of glucocorticoids.

## 1. Introduction

Immune abnormalities and inflammatory responses are the pathogenic mechanisms of many kidney diseases and are finely regulated by the local microenvironment [1,2]. Among them, renal proximal tubular epithelial cells (PTECs) are cells that have both immunoregulatory and biological functions [3]. Under the induction of pathological factors, their excessively produced inflammatory mediators [4,5,6] and complements [7,8] play an important role in the local inflammatory immune response.

Glucocorticoids are widely used in the treatment of immune-mediated kidney diseases (such as primary nephrotic syndrome, multiple glomerulonephritis, and some interstitial nephritis) due to their exact and powerful anti-inflammatory and immunosuppressive effects [9,10,11]. Among them, methylprednisolone (MP) is one of the most representative drugs that belong to intermediate-acting glucocorticoids and has a short biological half-life [12]. MP has anti-inflammatory, immunosuppressive, anti-allergic, anti-shock, and other pharmacological effects. Its immunosuppressive strength is better than that of other similar drugs, and its anti-inflammatory strength is 5 and 1.25 times that of hydrocortisone and prednisolone, respectively. Moreover, the water-sodium retention of MP is less than that of the two abovementioned glucocorticoids.

However, due to the nonspecific distribution of glucocorticoids in the body, large-dose systemic administration of glucocorticoids can produce a variety of systemic side effects, triggering iatrogenic adrenal hyperfunction syndrome; inducing and exacerbating infections; causing gastrointestinal symptoms, ulcers, cardiovascular system symptoms and so on [13,14]. Solving the systemic side effects caused by glucocorticoids in the treatment of kidney disease has always been a challenge to be addressed.

Local administration of glucocorticoids is a commonly used method and has been used clinically for the treatment of nonrenal diseases to reduce systemic side effects [15,16,17]. Because it is difficult for the kidneys to achieve local administration, researchers are also trying to use carriers to deliver glucocorticoids to the kidney, and some exploratory studies have been carried out, such as those with prednisone- 50% N-acetylated low-molecular-weight chitosan, prednisolone carbamate–glucosamine, prednisolone–folate [18,19,20]. These conjugates showed good renal targeting, in which the prednisolone–folate conjugate significantly reversed disease progression in animal models of renal ischemia–reperfusion injury. However, this design still had shortcomings. The carrier used in this conjugate was a small molecule, and the related receptors, folic acid receptors, were also widely distributed in other parts of the body, which may reduce the specific renal targeting of the drug [21,22].

In this article, we first report the methylprednisolone–lysozyme (MPS–LZM) conjugate, which was obtained by covalently linking MP and the renal targeting carrier LZM through a linker containing an ester bond, aiming to utilize the properties of LZM to achieve renal-specific delivery of the glucocorticoid. LZM is an ideal renal-targeting carrier for several reasons: first, as a low-molecular-weight protein, it can freely filter the glomeruli and be effectively absorbed by PTECs of the kidneys [23], but other parts of the body do not have this specific structure, and it has been widely used in other drug–LZM conjugates for the treatment of renal disease [24,25]; secondly, LZM has high safety and can be injected and taken orally. As a drug and food additive, it has been widely used in food and medicine for antiseptic and antibacterial purposes [26,27,28,29]. In addition, the combination of these two already-marketed drugs is conducive to increasing the possibility of it being a new drug. For this, we synthesized MPS–LZM and evaluated some of its properties in vitro and in vivo, including renal cell uptake, cytotoxicity in vitro, and pharmacokinetics and biodistribution in vivo, to clearly study the design feasibility of the MPS–LZM conjugate and develop a basis for further research on the treatment of immune-mediated kidney diseases.

## 2. Results and Discussion

### 2.1. Design of MPS–LZM

**Figure 1:**
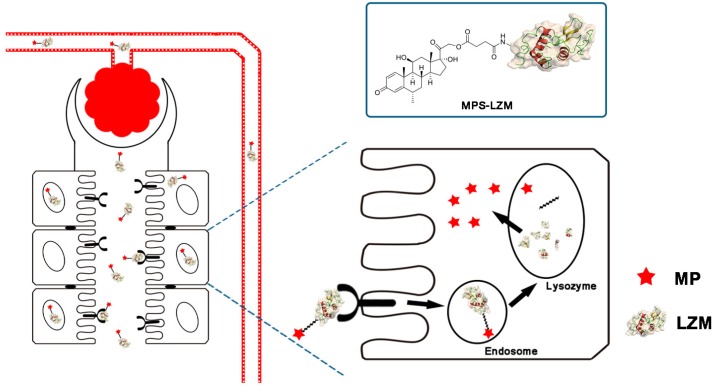
Schematic diagram of methylprednisolone–lysozyme (MPS–LZM) and renal-targeting drug delivery. Nephrons are the functional part of the kidneys, each consisting of a glomerulus and the renal tubule. MPS–LZM can freely filter the glomerulus and be effectively absorbed by proximal tubular epithelial cells (PTECs) of the kidneys.

By covalently linking MP and the renal-targeting carrier LZM through a linker containing an ester bond, we designed an MPS–LZM conjugate for the first time (Figure 1). First, the conjugate utilized the properties of lysozyme to achieve renal-specific delivery of MP, and increased its distribution in kidneys, thereby achieving the purpose of reducing toxicity and increasing efficiency. As a low-molecular-weight protein, LZM can freely filter the glomerulus and be effectively absorbed by PTECs of the kidney. The most abundant megalin receptors are expressed in PTECs, which can effectively recognize LZM and thereby achieve endocytosis [30,31,32]. Because the vascular endothelium of other organs does not have a structure that small-molecular-weight proteins can permeate, this makes the renal targeting of LZM specific. Secondly, after being endocytosed by PTECs, the LZM enters the lysosome through the endosome [31]. Then, LZM is degraded, and the ester bond in the linker is broken. Thus, the active molecule, MP, is released. Finally, the released MP exerts an immunosuppressive effect on tubule epithelial cells or spreads to the glomerular site through local diffusion, thereby improving the treatment of related immune-mediated renal diseases.

### 2.2. Synthesis and Characterization of MPS–LZM

**Figure 2:**
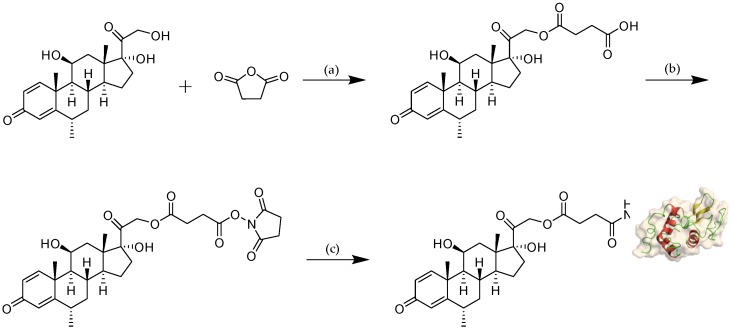
Reaction scheme for the synthesis of MPS–LZM. Reagents and conditions: (**a**) 4-dimethylaminopyridine (DMAP), pyridine, rt, overnight, 89%; (**b**) N-hydroxysuccinimide (NHS), N,N’-dicyclohexylcarbodiimide (DCC), Tetrahydrofuran (THF), rt, 6 h; (**c**) LZM, PBS (pH 7.5)/N,N-dimethylacetamide (DMA), rt, 8 h. rt: room temperature.

**Figure 3:**
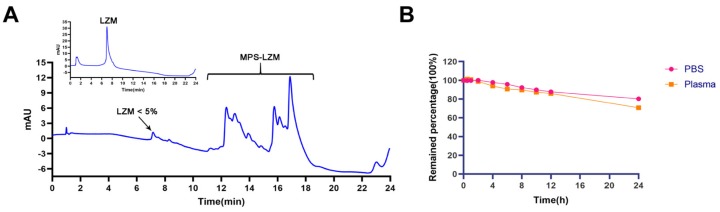
(**A**) Hydrophobic interaction chromatography (HIC)–HPLC analysis of MPS–LZM. HIC allowed resolution of the conjugate with LZM. LZM content is less than 5% in the conjugate; (**B**) The stability of MPS in PBS (pH 7.4) and plasma at 37 °C for 24 h. Data represent mean ± SD (*n* = 3).

The reaction scheme of MPS–LZM is presented in Figure 2. We first tried to directly couple MPS and LZM, but the efficiency was very low. Then, we separated the MPS–NHS ester and reacted it with LZM, which made the preparation very simple, and the reaction conditions were mild. Finally, the conjugate was analyzed by quadrupole Fourier transform ion cyclotron resonance mass spectrometry (Q–FT–ICR MS), which proved the success of the coupling (Figure S1). At the same time, the residual LZM content in the conjugate was detected by hydrophobic interaction chromatography (HIC)–HPLC. The test result is shown in Figure 3A, which shows that the residual LZM was less than 5%. To prove the reliability of the coupling method, we carried out quality studies on the coupling products of multiple batches (Figure S2). The results show that the coupling process of the final product had good reproducibility and could maintain uniformity and stability between batches. The endotoxin content of the conjugate was also controlled, which was less than 5 EU/mg, as determined by the tachypleus amebocyte lysate (TAL) method and met the quality standards for further efficacy studies. Two methods were used to determine the average coupling ratio: UV spectrophotometry and the hydrolysis method (confirmed by BCA protein analysis and measurement of the coupled MP by HPLC). The results of the two methods were basically the same, indicating a 1.1:1 coupling ratio of MP to LZM. To verify the uptake of the conjugate in vitro and its biological distribution in vivo, we also synthesized FITC-labeled MPS–LZM.

MPS–LZM is a typical prodrug, and the evaluation of a prodrug is first to satisfy a certain plasma stability and to be effectively released at the target site. To this end, we evaluated the stability of MPS in phosphate buffer saline (PBS) and plasma. After incubation in PBS (pH 7.4) and human plasma for 24 h at 37 °C, the remaining percentages of MPS were 80.32% and 70.80%, respectively (Figure 3B), and the stability fully met the requirements.

### 2.3. Cellular Uptake of MPS Proximal Tubular Epithelial Cells (PTECs) LZM

**Figure 4:**
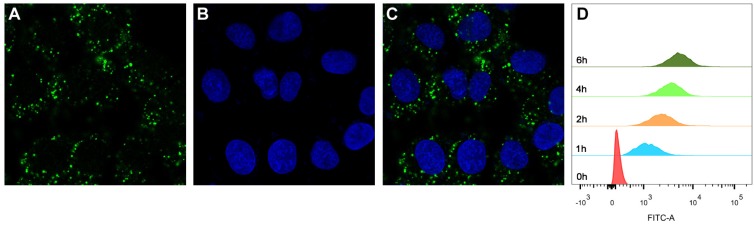
Cellular uptake of FITC-labeled MPS–LZM by HK-2 cells. Confocal micrographs of (**A**) FITC-labeled MPS–LZM, (**B**) DAPI staining nucleus, and (**C**) merged of FITC-labeled MPS–LZM and DAPI. Cells were exposed to FITC-labeled MPS–LZM for 2 h at 37 °C; (**D**) Flow cytometric analysis of HK-2 cells exposed to FITC-labeled MPS–LZM and control cells.

Since PTECs are the main cell type that plays the role of reabsorption after glomerular filtration [33,34], whether MPS–LZM can be effectively taken up by PTECs is the basis of this research. We first evaluated the endocytosis of MPS–LZM by PTECs using FITC-labeled MPS–LZM. As shown in Figure 4A–C, FITC-labeled MPS–LZM showed obvious endocytosis after incubation with HK-2 for 2 h at 37 °C.

To evaluate whether the cellular uptake of the conjugate was time-dependent, we measured the fluorescence intensity of HK-2 cells after different incubation times with FITC-labeled MPS–LZM using flow cytometry. As shown in Figure 4D, the fluorescence intensity became stronger with time, which indicates that the cellular uptake of the conjugate was time-dependent over a certain time frame. The covalent binding of small-molecule drugs did not affect the LZM binding and endocytosis characteristics of HK-2 cells, which indicates that the conjugate could be effectively endocytosed by renal PTECs and that MP was then released intracellularly.

### 2.4. In Vitro Cytotoxicity of MPS-LZM

**Figure 5:**
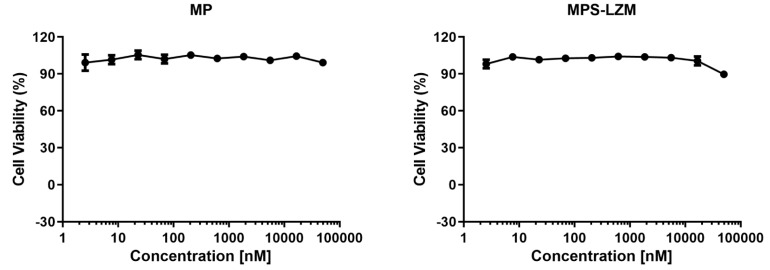
Cell viability of HK-2 cells after 24 h exposure to 0–50 μM methylprednisolone (MP) and MPS–LZM. Untreated HK-2 cells were used as control and set at 100%. Error bars represent the standard deviation from duplicate determinations, and results are shown as mean ± SD. No obvious toxicity was observed for any of the evaluated compounds.

The renal proximal tubule is the most common site of drug nephrotoxicity [35], and PTECs are the target cells of MPS–LZM, so it is necessary to evaluate the safety of the conjugate for PTECs. The cytotoxic effect of MPS–LZM on cell proliferation was evaluated in HK-2 cells. As shown in Figure 5, 0–50 μM MP and MPS–LZM were incubated with HK-2 cells for 24 h without showing significant cytotoxic effects (IC_50_ > 50 μM), which initially met the requirements of MPS–LZM for further research.

### 2.5. In Vivo Pharmacokinetics of MPS–LZM

#### 2.5.1. Plasma Disappearance of MPS–LZM

**Figure 6:**
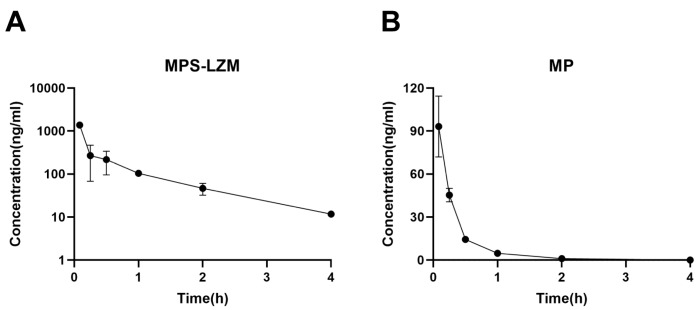
Plasma disappearance of (**A**) MPS–LZM and (**B**) MP after intravenous administration. Data represent mean ± SD (*n* = 6).

The retention of MPS–LZM in the circulatory system is evaluated by measuring the MP content in plasma. After intravenous administration, MPS–LZM is rapidly eliminated in the circulatory system (Figure 6A), as expected, indicating its rapid accumulation in the kidney. Despite rapid elimination, the presence of conjugate could still be detected in the circulatory system at 12 h. A small amount of free MP could also be detected in the circulatory system, but its exposure (45.52 h*ng/mL of AUC_0–t_) only accounted for 7.8% of the total MP corresponding to the conjugate (632.72 h*ng/mL of AUC_0–t_), possibly due to the nonspecific release of MP from the conjugate in the circulatory system or the reentry of the released MP from the kidneys into blood. Compared with the disappearance data of MP in plasma (Figure 6B), the conjugate showed a significant difference in the volume of distribution (Vz). As shown in Table 1, the pharmacokinetic parameters are calculated by fitting the data to a non-compartment model. The distribution volume of MP was 3.3 times that of the conjugate, reflecting the difference in penetration in cells and tissues, as well as in protein binding in plasma and tissues, between MP and the macromolecular conjugate [36]. Unlike small molecules, because macromolecule conjugate cannot passively diffuse across the membranes, the distribution of the conjugate is limited to circulation and the intercellular substance, which promotes its filtration through the glomerulus and accumulation in the kidneys. This phenomenon is consistent with other drug–LZM conjugates [37].

#### 2.5.2. Renal Accumulation of MPS–LZM

**Figure 7:**
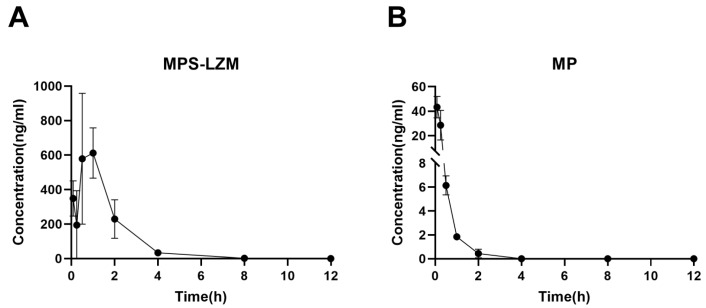
Renal accumulation of of (**A**) MPS–LZM and (**B**) MP after intravenous administration. Data represent mean ± SD (*n* = 6).

Achieving effective accumulation of MPS–LZM in the kidneys is the purpose of developing a renal-targeting conjugate. Therefore, we evaluated the renal accumulation of the conjugate in mice using liquid chromatographic/mass spectrometric (LC/MS). Data were fitted with a non-compartment model, and the pharmacokinetic parameters of the renal data are summarized in Table 2. Corresponding to the rapid elimination of MP from kidneys, MPS–LZM accumulated rapidly in the kidneys (Figure 7). The conjugate could be detected in the kidneys 5 min after intravenous administration and the maximum drug level C_max_ (612.19 ng/mL) was observed in the kidneys at 1 h. The rapid accumulation in the kidneys showed good agreement with the rapid elimination in plasma. Simultaneously, comparing the MRT_(0–t)_ of MPS–LZM (1.44 h) with MP (0.29 h), the conjugate showed a relatively prolonged renal retention, and it could still be detected in kidneys up to 8 h after administration, which indicates that the conjugate could potentially enhance the therapeutic effect.

#### 2.5.3. Intrarenal Release of MP

**Figure 8:**
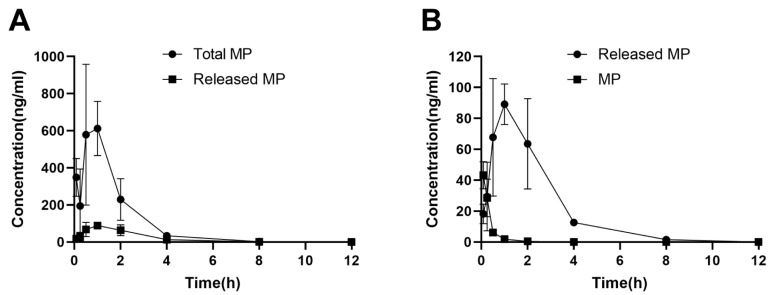
(**A**) Total and released MP levels in the kidneys after administration of MPS–LZM; (**B**) MP levels in the kidneys after administration of MPS–LZM and MP. Data represent mean ± SD (*n* = 6).

MP released from MPS–LZM in PTECs may be necessary for the activity of the conjugate. We therefore measured total MP and released MP levels in kidneys after intravenous administration of MPS–LZM (Figure 8A). The released MP was detected within 5 min after administration and reached a maximum renal level C_max_ at 1 h. At the same time, we compared the levels of MP released from MPS–LZM with those obtained after administration of MP in the kidneys (Figure 8B). Although the initial concentration was higher after administration of MP, it was rapidly eliminated from the kidneys. In contrast, MPS–LZM could provide relatively long-term intrarenal MP levels. Therefore, compared with MP, MPS–LZM resulted in a higher active MP exposure in the kidneys, and the AUC_0–t_ increased from 17.59 to 242.18 h*ng/mL (14 times), which indicates that MPS–LZM is likely to exhibit a better therapeutic effect in the treatment of immune-mediated renal diseases.

### 2.6. Biodistribution of MPS-LZM in Vivo

**Figure 9:**
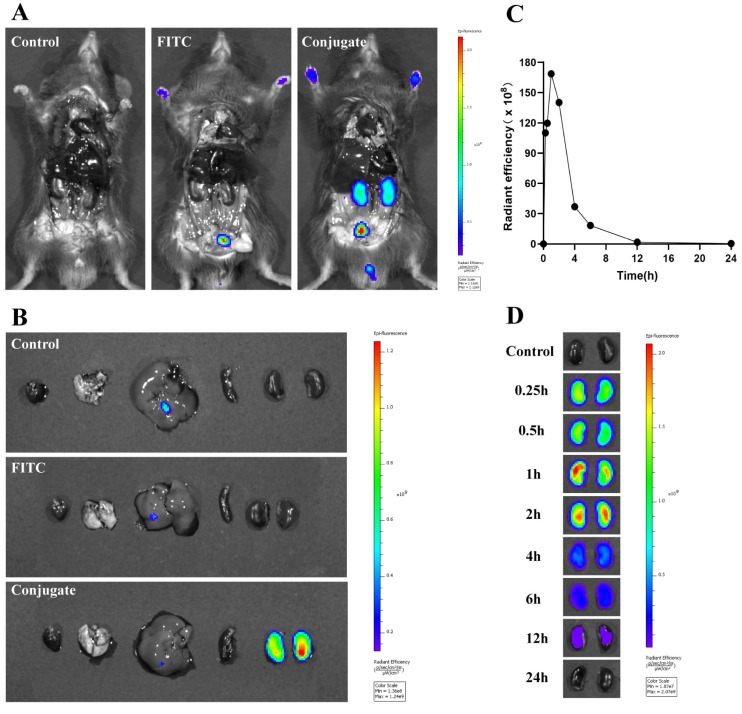
Biodistribution of FITC-labeled MPS–LZM in vivo. (**A**) Representative whole body distribution of FITC-labeled MPS–LZM or equivalent free FITC 1 h after tail vein injection; (**B**) Excised main organs 1 h after tail vein injection were collected and photographed; (**C**) Quantitative analysis of fluorescent signal of excised kidneys at different time intervals; (**D**) Excised kidneys at different time intervals after injection were compared and photographed.

To characterize the distribution of the conjugate more intuitively in vivo, mice were administered FITC-labeled MPS–LZM. As shown in Figure 9A,B, the kidneys and bladder were the only tissues that could detect fluorescence, and the fluorescence was not detected in other tissues, indicating that coupling with MP did not alter the renal targeting properties of LZM. The FITC-labeled conjugate could be taken up by the kidneys within 15 min and reach maximum aggregation in the kidneys at 1 h. At the same time, the presence of fluorescence was still visible for a relatively long time (at least 12 h, Figure 9C,D). These qualitative results are basically consistent with the quantitative measurements of pharmacokinetics of MPS–LZM. Based on the rapid renal distribution and the specific renal retention time of MPS–LZM, it could be particularly suitable for the continuous treatment of certain immune-mediated renal diseases by intravenous drip in clinical practice.

## 3. Materials and Methods

### 3.1. Chemistry

MP was purchased from Adamas-beta (Shanghai, China), Hen egg white lysozyme was purchased from Sigma-Aldrich (Canada). Acetonitrile (HPLC grade) was purchased from Thermo Fisher Scientific (Shanghai, China). The other chemicals were of analytical grade and used without additional purification. Reactions were followed by thin-layer chromatograph (Dexin Biotechnology Co., Ltd., Yantai, China). Visualization was accomplished with 254 nm UV light.

The structures of the products were identified by ^1^H and ^13^C-NMR spectroscopy (JNM-ECA-400, Tokyo, Japan). Chemical shifts were reported in ppm and TMS was as the internal standard. Coupling constants J were given in Hertz. Spin multiplicities were reported as the following abbreviations: s (singulet), d (doublet), dd (doublet doublet), t (triplet), q (quadruplet), m (multiplet). The molecular weights of the products were measured by API 3000 triple-quadrupole mass spectrometer equipped with a turbo ion spray electrospray ionization (ESI) source (AB Sciex, Concord, ON, Canada). HPLC analysis was performed using an Agilent 1260 Series (California, CA, USA).

### 3.2. Synthesis of Methylprednisolone–Lysozyme and FITC-Labeled Methylprednisolone–Lysozyme

#### 3.2.1. Synthesis of MP Succinate (MPS)

MP (1.5 g, 4.01 mmoL) and 4-dimethylaminopyridine (DMAP, 137 mg, 1.12 mmol) were dissolved in 15 mL dry pyridine. The mixture was stirred at room temperature for 10 min and then succinic anhydride (520 mg, 5.20 mmol) was added. The reaction mixture was stirred at room temperature overnight. After completion of the reaction, 200 mL deionized water was added to the reaction solution and then adjusted the pH to about 4 with hydrochloric acid (10%) until solid precipitated out. Filtered and dried under reduced pressure to get the crude product as a white solid. Purification was performed by silica column chromatography in 25:1 dichloromethane/methanol (*v/v*) to give 1.69 g (89%) of MPS. ^1^H NMR (400 MHz, DMSO-d6) δ 12.26 (s, 1H), 7.32 (d, J = 10.1 Hz, 1H), 6.18 (dd, J = 10.1, 1.7 Hz, 1H), 5.82 (s, 1H), 5.41 (s, 1H), 5.08 (d, J = 17.6 Hz, 1H), 4.82–4.71 (m, 2H), 4.28 (s, 1H), 2.71–2.58 (m, 3H), 2.54–2.47 (m, 3H), 2.16–2.00 (m, 2H), 1.92–1.84 (m, 1H), 1.70–1.55 (m, 3H), 1.49–1.41 (m, 1H), 1.39 (s, 3H), 1.36–1.26 (m, 1H), 1.05 (d, J = 6.2 Hz, 3H), 0.85 (dd, J = 11.1, 3.2 Hz, 1H), 0.79 (s, 3H), 0.76–0.61 (m, 1H). ^13^C NMR (400 MHz, DMSO-D6) δ 205.25, 185.23, 173.56, 173.35, 171.74, 157.35, 126.77, 118.85, 88.64, 68.35, 67.72, 55.97, 51.04, 47.14, 44.09, 42.98, 33.17, 32.53, 30.84, 28.73, 28.51, 23.54, 21.42, 17.71, 16.61. ESI m/z: C_26_H_34_O_8_ calculated 474.2254; found 473.2175 (M−H)^−^.

#### 3.2.2. Synthesis of MPS NHS Ester

A solution of MPS (150 mg, 0.32 mmol), NHS (48 mg, 0.41 mmol) and DCC (98 mg, 0.47 mmol) in THF (6 mL) was stirred at room temperature for 6 h. The reaction mixture was cooled to −20 °C for 2 h and then filtered to remove the insoluble dicyclohexylurea (DCU). The filtrate was evaporated under reduced pressure and the residue was reconstituted in 50 mL dichloromethane. The organic layers were washed with dilute NaHCO_3_ and brine, dried over anhydrous Na_2_SO_4_, and evaporated under reduced pressure to give the crude product. Purification was performed by stirring in diethyl ether and relatively pure product was obtained through filtration, which was used for the next step directly without further purification.

#### 3.2.3. Synthesis of MPS–LZM

LZM (60 mg, 4.20 μmol) was dissolved in 7 mL PBS (0.1 M, pH 7.5) by stirring gently. MPS NHS ester (9.6 mg, 16.80 μmol) in 1.2 mL dimethylacetamide (DMA) was added dropwise and the reaction mixture was stirred for 8 h at room temperature. After centrifugation (4000 rpm, 10 min), the supernatant was purified by using a Sephadex G-25 column on AKTA pure 25 chromatographic system to remove uncoupled MPS NHS ester and other low molecular residues. The protein fraction of the eluents was dialyzed against distilled water at 4 °C for 24 h and subsequently lyophilized. HIC–HPLC analysis of MPS–LZM was performed on a TSKgel Butyl-NPR column (2.5 µm, 4.6 × 100 mm; Tokyo, Japan) using a 0.1 M KH_2_PO_4_ water (pH 7.0)/1.8 M (NH_4_)_2_SO_4_ water (pH 7.0) gradient.

#### 3.2.4. Synthesis of FITC Labeled MPS–LZM

MPS–LZM (23 mg, 1.50 mmol) was dissolved in 13 mL PBS (pH 7.5) by stirring gently. An equivalent amount of FITC in 1.5 mL dimethyl sulfoxide (DMSO) was added dropwise and the reaction mixture was stirred for 3 h at room temperature away from light. After centrifugation (4000 rpm, 10 min), the supernatant was purified by using a PD10 column to remove uncoupled FITC. The appropriate fractions were concentrated by centrifugal ultrafiltration and sterile filtered through a 0.2-µm filter under sterile conditions to get FITC labeled MPS–LZM.

### 3.3. Characterization of MPS–LZM

MPS–LZM was characterized for protein content (Pierce™ BCA Protein Assay Kit, Thermo Scientific, USA) and the amount of conjugated MP. For the latter assay, MP was released from the conjugate by hydrolysis and then analyzed by HPLC, as previously described, with modifications [38]. Briefly, samples were dissolved in water and subsequently incubated with an equivolume of 0.1 M NaOH for 10 min, neutralized with equimolar HCl, diluted 5× with methanol and centrifuged. Released MP was determined on an Agilent Eclipse XDB-C18 column (5 μm, 4.6 × 250 mm; California, CA, USA), and the mobile phase was composed of 32% acetonitrile and 68% buffer (50 mM KH_2_PO_4_ water). MP was detected at 252 nm, and the total analytical time was 20 min for each run.

To characterize the stability of the conjugate, MPS was incubated in PBS (pH 7.4) and 50% human plasma for 24 h. Briefly, 100 μM MPS in PBS (pH 7.4) or 50% human plasma, containing 10% DMSO, were transferred to a centrifuge tube, and incubated at 37 °C. At predetermined time points (0, 0.5, 1, 2, 4, 6, 8, 10, 12, 24 h), 200-μL samples were taken out and diluted with methanol (1 mL). The mixtures were then vortexed for 5 min and centrifuged at 5000× *g* for 20 min. The supernatants were analyzed by HPLC on an Agilent Eclipse XDB- C18 column (5 μm, 4.6 × 150 mm; California, CA, USA) using 35% acetonitrile/65% buffer (50 mM KH_2_PO_4_ water). MPS was detected at 252 nm, and the total analytical time was 10 min for each run.

### 3.4. In Vitro Cell Studies

The immortalized human renal proximal tubule epithelial cell line (HK-2) (American Type Culture Collection, Manassas, VA, USA) was cultured in DMEM supplemented with 10% (*v/v*) fetal bovine serum, penicillin (100 U/L), and streptomycin (100 μg/mL) at 37 °C in a humidified atmosphere containing 5% CO_2_.

#### 3.4.1. In vitro Cytotoxicity of MPS–LZM and MP

HK-2 cells were seeded (2000 cells/well in a total volume of 30 μL complete medium) in 384-well microplates 24 h prior to the addition of the samples. The cells were then incubated with 0–50 μM MPS–LZM and MP for 24 h under normal culture conditions. The cytotoxicity of the samples was established using the CellTiter-Glo assay kit (CTG). IC_50_ values were calculated using GraphPad Prism 8 software.

#### 3.4.2. In vitro Endocytosis of MPS–LZM

HK-2 cells were seeded (2 × 10^4^ cells/well in a total volume of 400 μL complete medium) in chamber slides and incubated for 12 h. Cells were then incubated with FITC-labeled MPS–LZM at a final dose of 0.4 mg/mL under normal culture conditions. After the conjugate was incubated with HK-2 cells for 2 h, 200 μL PBS was used to wash cells three times to remove any extracellular drug compounds, and fluorescence images were taken using a laser confocal microscope (ZEISS LSM 880, Germany).

#### 3.4.3. In vitro Uptake Assay of MPS–LZM

The cultured HK-2 cells were seeded in 12-well Costar^®^ plates (2 × 10^5^ cells/well) and incubated for 24 h. Then, the cells were exposed to FITC-labeled MPS–LZM, which was diluted in DMEM at a final dose of 0.3 mg/mL for 1, 2, 4, and 6 h at 37 °C.

To quantify the cellular uptake of FITC-labeled MPS–LZM, flow cytometric analysis (Cytomics FC 500, Beckman Coulter, USA) was performed after the test samples were aspirated and the cells, trypsinized with trypsin/EDTA and washed twice with PBS, were resuspended in 1 mL of PBS. The fluorescence of 10,000 events was determined, and the data were analyzed by using FlowJo VX software. Untreated HK-2 cells served as a negative control.

### 3.5. Animals

In vivo experiments were performed in normal male C57BL/6J mice obtained from Ling Chang Biological Technology Co., Ltd. (Shanghai, China). Mice 7–8 weeks of age, with a bodyweight of 18–30 g were ordered and used within two weeks after arrival. Mice were housed in cages under a 12 h light and 12 h dark cycle and given food and water ad libitum. All procedures related to animal selection, handling, and treatment were performed according to the Medicilon Preclinical Research (Shanghai) LLC Study Protocol and Standard Operating Procedures (SOPs). All animals in this study were treated in accordance with the Guide for the Care and Use of Laboratory Animals.

### 3.6. Pharmacokinetics and Renal Distribution of MPS–LZM

The pharmacokinetics and renal distribution of MPS–LZM were studied in mice and compared with the pharmacokinetics and renal distribution of MP as previously described, with modifications [24]. Male mice were treated with a single intravenous injection of either 10 mg/kg MPS–LZM or an equimolar dose of MP. Six mice were employed in each group, and animals were sacrificed at the indicated time points. Blood samples were collected via the jugular vein, centrifuged at 8000 rpm for 6 min at 2–8 °C and stored frozen at −80 °C until the bioanalysis as described below. The kidneys were also collected and stored frozen at −80 °C until further processing as described below.

### 3.7. LC–MS/MS Analysis of MP Concentrations in Plasma and Tissues

#### 3.7.1. Mice Treated with a Single Injection of MP

MP concentrations in plasma and kidneys were measured. Standard calibration samples were prepared in a concentration range of 1–1000 ng/mL MP in blank mouse plasma and 5–5000 ng/g in blank kidney homogenate. Plasma samples were processed undiluted. Ten-microliter plasma samples were mixed with 200 μL Warfarin internal standard solution (1000 ng/mL in methanol). The mixture was vortexed and then centrifuged for 5 min at 14,000 rpm, and 180 μL aliquots of the supernatant were transferred to 96-well plates and injected (3 μL) for LC–MS/MS analysis as described below. Kidney samples were homogenized first with 50% methanol/water at a final concentration of 0.1 g tissue/mL. Homogenized samples (30 μL) were mixed with 300 μL Warfarin internal standard solution (1000 ng/mL in methanol), and subsequent treatment was the same as that of the plasma samples.

The LC system comprised of a Waters (Waters Corporation, UAS) ultraperformance liquid chromatography (UPLC) instrument equipped with an ACQUITY UPLC binary solvent manager, ACQUITY UPLC autosampler, ACQUTIY UPLC sample organizer and ACQUITY UPLC column heater. An MS analysis was performed using an API 4000 (triple quadrupole) instrument from Applied Biosystems/MDS Sciex with an ESI source. Samples were separated on an ACQUITY UPLC BEH C18 column (50 × 2.1 mm, 1.7 μm). The autosampler temperature was maintained at 4 °C, and the column temperature was maintained at 50 °C. The mobile phase consisted of 0.1% formic acid in water (A) and 0.1% formic acid in methanol (B). The flow rate was 0.5 mL/min, and the total running time was 3.0 min. The amount of solvent B was increased from 20% to 90% during the first 0.4 min after injection, followed by flushing the column for 0.4 min with 90% solvent B and reconditioning the column at 20% solvent B for 0.4 min. The ion spray voltage was maintained at 4.5 kV, and the heated-capillary temperature was set at 450 °C. Dwell times were 150 ms for MP and 100 ms for the internal standard. MP was monitored at m/z 419.3→343.0, and the internal standard was monitored at m/z 307.0→249.7.

#### 3.7.2. Mice Treated with a Single Injection of MPS–LZM

Total MP (reflecting the sum of MP bound to LZM and LZM-released MP) levels were measured in the plasma and kidneys. In addition, released MP levels were also measured in plasma and kidneys.

For the analysis of total MP concentrations in the plasma and kidneys, 50 μL plasma samples or kidney homogenate was added to 5 μL Warfarin internal standard solution (1000 ng/mL) and 0.1 M NaOH (50 μL). These mixtures were then vortexed and hydrolyzed at room temperature for 30 min, and then 0.1 M HCl (50 μL) was added for neutralization.

MP was extracted using tert-butyl methyl ether (TBME). After the addition of TBME, the samples were vortexed and centrifuged for 10 min at 12700 rpm. The supernatant, TBME, was dried under a nitrogen stream at 40 °C, and the residue was reconstituted in 250 µL 50% methanol/water solution. Supernatants were then analyzed by LC–MS/MS, as described above.

### 3.8. Biodistribution and Intrarenal Fate of FITC-Labeled MPS–LZM

The mice received 10 mg/kg FITC-labeled MPS–LZM and an equivalent amount of free FITC in PBS intravenously as previously described, with modifications [24]. The mice were sacrificed at 1 h, and the intestines were immediately removed to expose the main organs before imaging. The heart, liver, spleen, lungs, and kidneys were also collected for imaging on an IVIS^®^ Spectrum (PerkinElmer, USA).

To observe how long MPS–LZM can be retained in the kidneys, mice were injected with FITC-labeled MPS–LZM and sacrificed at predetermined time points (0.25, 0.5, 1, 2, 4, 6, 12, 24 h). The kidneys were then collected and stored at −80 °C away from light. After all the kidneys were collected at different time points, fluorescence imaging was performed on an IVIS^®^ Spectrum (PerkinElmer, USA).

## 4. Conclusions

We have developed an MPS–LZM conjugate that can specifically deliver MP with anti-inflammatory and immunosuppressive functions to PTECs to regulate the immune microenvironment. Both in vivo and in vitro experiments have demonstrated the renal targeting effect of the conjugate. Future research will further evaluate its application in animal models related to immune-mediated renal diseases, such as animal models of interstitial nephritis and IgA nephropathy, to prove the feasibility of renal targeting strategies and promote the clinical application of the renal targeting conjugate.

## Figures and Tables

**Table 1:** Pharmacokinetic parameters of MP and MPS–LZM in plasma after a single intravenous injection.

Pharmacokinetic Parameter	MP	MPS–LZM
t_1/2_ (h)	0.44	2.29
Vz (mL/kg)	4811.80	1454.28
MRT_(0–t)_ (h)	0.30	0.87

**Table 2:** Pharmacokinetic parameters of MP and MPS–LZM in kidneys after a single intravenous injection.

Pharmacokinetic Parameter	MP	MPS–LZM
t_1/2_ (h)	0.49	1.09
T_max_ (h)	0.08	1.00
C_max_ (ng/mL)	43.27	612.19
AUC_(0–t)_ (h*ng/mL)	17.59	1238.65
MRT_(0–t)_ (h)	0.29	1.44
		
Released MP in the kidney		
AUC_(0–t)_ (h*ng/mL)	—	242.18

## Supplementary Materials

Supplementary materials can be found at https://www.mdpi.com/1422-0067/21/6/1922/s1.

## Abbreviations

MP	Methylprednisolone
MPS-LZM	Methylprednisolone–Lysozyme
PTECs	Proximal tubular epithelial cells
HIC	Hydrophobic interaction chromatography
HPLC	High performance liquid chromatographic
TAL	tachypleus amebocyte lysate
LC/MS	Liquid chromatographic/Mass spectrometric
NMR	Nuclear magnetic resonance
