# Supplementary figures and images for: Design, Synthesis, and Renal Targeting of Methylprednisolone-Lysozyme
Source: Int J Mol Sci. 2020 Mar 11;21(6):1922. doi: 10.3390/ijms21061922 (PMC7139590; doi:10.3390/ijms21061922)

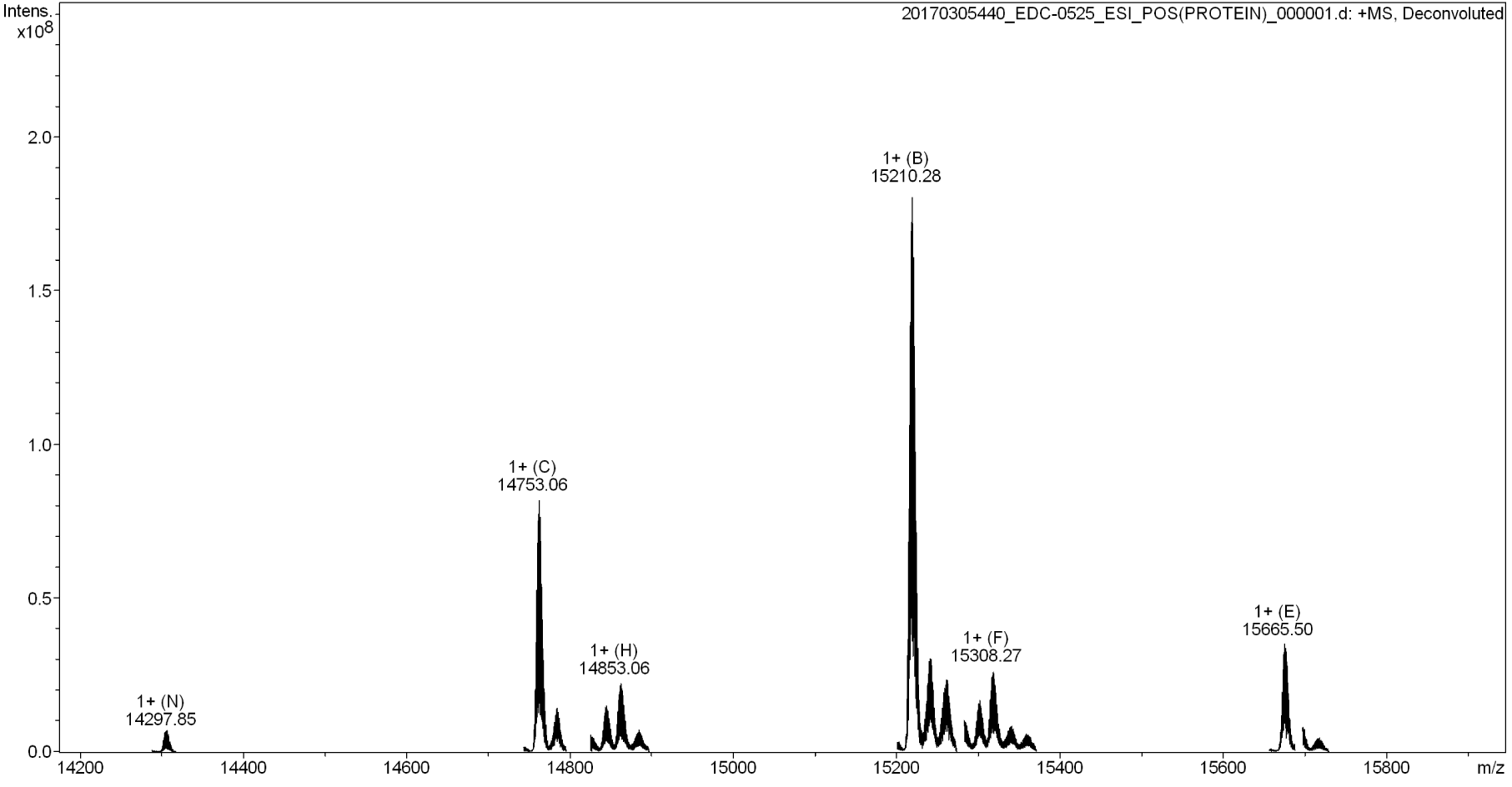

Supplement: Supplementary file 1 [file ijms-21-01922-s001.zip › Supplementary materials/Figure S1.tif]

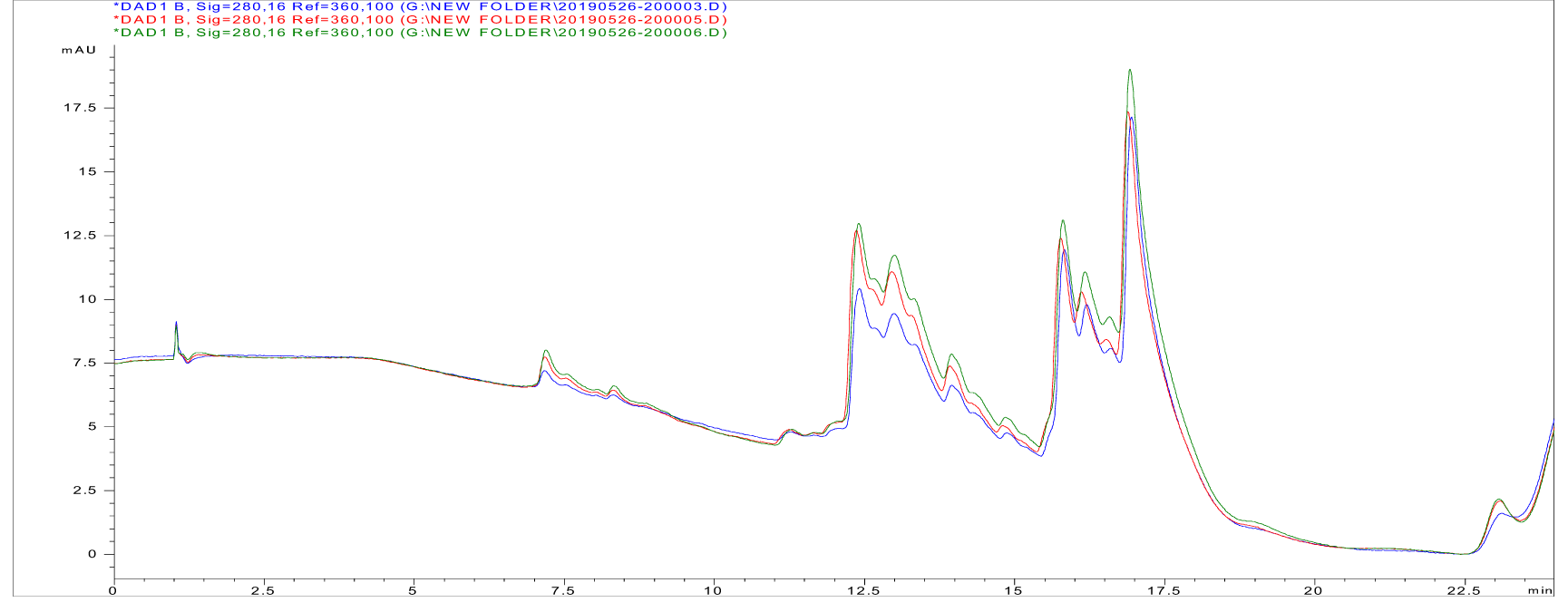

Supplement: Supplementary file 1 [file ijms-21-01922-s001.zip › Supplementary materials/Figure S2.tif]

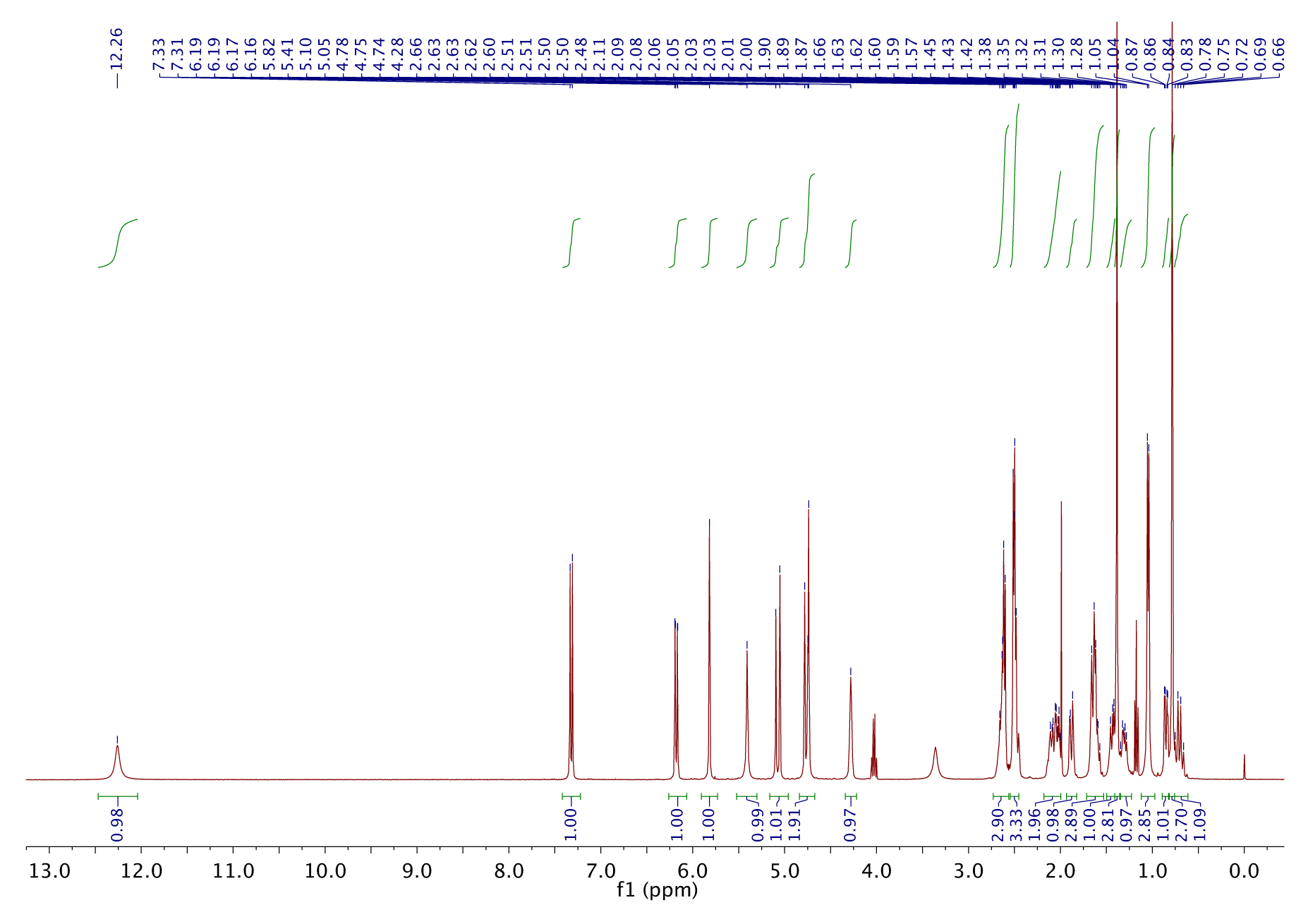

Supplement: Supplementary file 1 [file ijms-21-01922-s001.zip › Supplementary materials/Figure S3.tif]

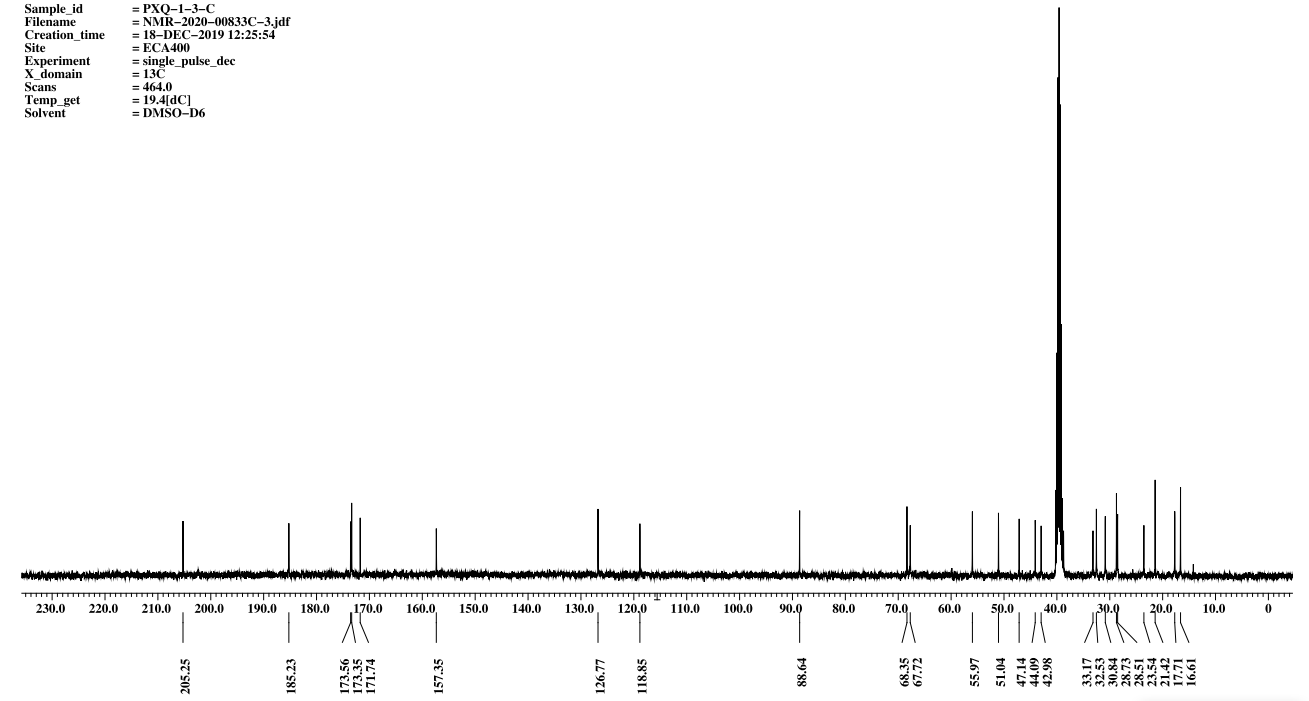

Supplement: Supplementary file 1 [file ijms-21-01922-s001.zip › Supplementary materials/Figure S4.png]

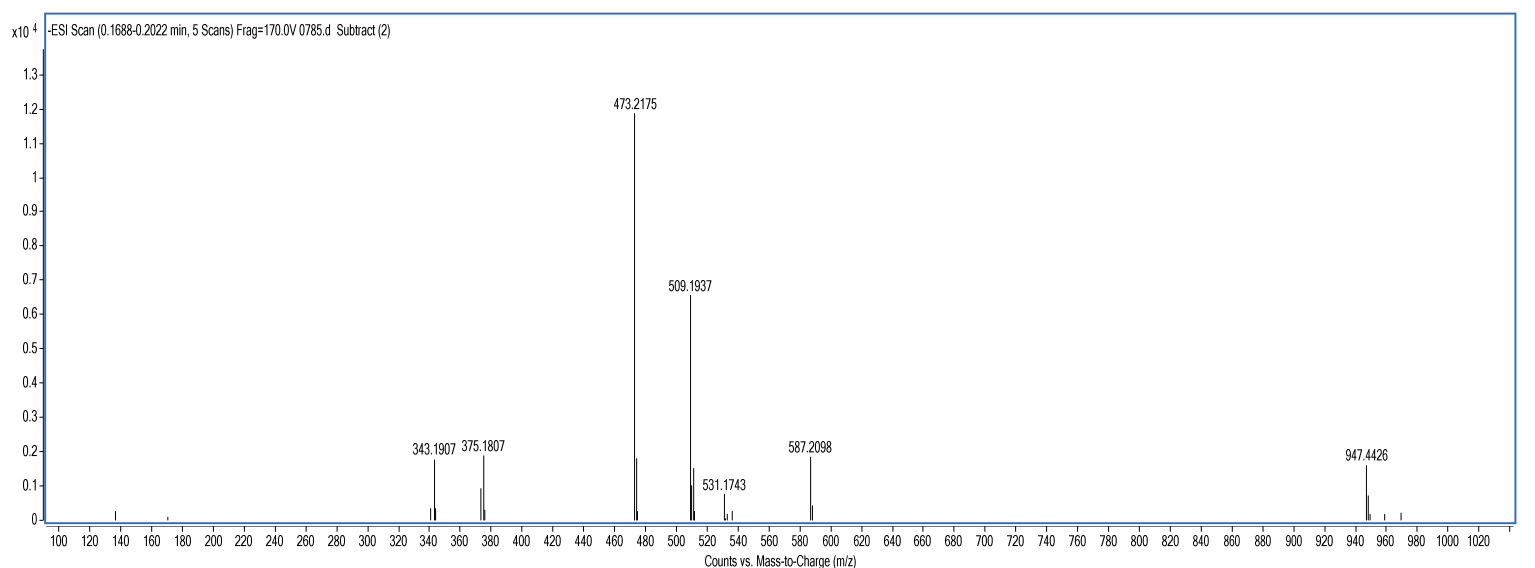

Supplement: Supplementary file 1 [file ijms-21-01922-s001.zip › Supplementary materials/Figure S5.png]
